# The effect of rose damascene extract on anxiety and sexual function of breastfeeding women: a randomized controlled trial

**DOI:** 10.3389/fmed.2024.1466341

**Published:** 2024-12-05

**Authors:** Gohar Akbarzadeh, Parvin Abedi, Shayesteh Jahanfar, Hossein Sadeghi Mansurkhani, Ahmad Fakhri, Elham Maraghi

**Affiliations:** ^1^Midwifery Department, Reproductive Health Promotion Research Center, Ahvaz Jundishapur University of Medical Sciences, Ahvaz, Iran; ^2^Midwifery Department, Menopause Andropause Research Center, Ahvaz Jundishapur University of Medical Sciences, Ahvaz, Iran; ^3^Department of Public Health and Community Medicine, Tufts School of Medicine, Boston, MA, United States; ^4^Department of Physiology and Pharmacology, School of Medicine, Medicinal Plants Research Center, Yasuj University of Medical Sciences, Yasuj, Iran; ^5^Department of Psychiatry, Ahvaz Jundishapur University of Medical Sciences, Ahvaz, Iran; ^6^Department of Biostatistics, Ahvaz Jundishapur University of Medical Sciences, Ahvaz, Iran

**Keywords:** sexual function, breastfeeding, state anxiety, trait anxiety, reproductive

## Abstract

**Background:**

Sexual dysfunction is prevalent among breastfeeding women.

**Objective:**

This study was designed to evaluate the effect of Rose damascene extract on sexual function and anxiety of breastfeeding women.

**Materials and methods:**

This was a randomized controlled trial. This study was conducted on 87 breastfeeding women who received either Rose damascene (*n* = 44) or placebo (*n* = 43) for eight weeks. The primary outcomes were sexual function and anxiety. A demographic questionnaire, and obstetric questionnaire, the Female Sexual Function Scale (FSFI), and the Spielberger questionnaire were used to collect the data. Chi-square test, independent *t*-test, and ANCOVA were used to analyze the data.

**Results:**

In the intervention group, there was a significant increase in the scores of sexual desire, arousal, lubrication, orgasm, and sexual satisfaction, while the score of pain reduced significantly after eight weeks of intervention (*p* < 0.001). The mean total score of sexual function prior to the intervention was 19.61 ± 5.02 and 21.46 ± 3.08 in the intervention and control groups, respectively. After intervention, this score was improved in the intervention group compared to the control group (25.21 ± 1.62 vs. 21.82 ± 3, *p* < 0.001). The pre-intervention score of trait anxiety was 47.97 ± 4.68 and 48.44 ± 5.89 in the intervention and control groups, respectively, which was improved in the intervention group compared to the control group after intervention (51.63 ± 3.53 vs. 48.13 ± 5.57, *p* < 0.001). Although there was an improvement in the score of state anxiety in the intervention group compared to the control group, the difference was not statistically significant (43.31 ± 6.41 vs. 44.30 ± 8.87, *p* = 0.397).

**Conclusion:**

Rose damascene could significantly improve the sexual functions of breastfeeding women. It also decreased the trait anxiety but failed to improve the state anxiety significantly. Using this herb is recommended to enhance sexual performance in breastfeeding women.

**Clinical trial registration:**

https://www.irct.ir/trial/61375, identifier IRCT20211015052775N1.

## 1 Introduction

Sexual activity is defined as an emotional and physical connection with the spouse that plays an important role in the quality of life (1). According to Masters and Johnson, there are four phases of sexual responses in a normal sexual function, namely excitement, plateau, orgasm, and resolution (2). Sexual dysfunction is defined as an any shortcoming in the sexual responses or feeling of pain during intercourse (3).

Some demographic factors such as age and level of education have been reported as important factors in sexual life of a woman (4). Also, different periods in a woman’s life such as pregnancy (5), breastfeeding (6), and menopause (7) have important effects on sexual function and satisfaction.

Some factors that negatively influence sexual function during breastfeeding are lack of priority for sexual relations, limited communication with the husband, and low frequency of sexual relations (8). A study in Iran showed that the prevalence of sexual dysfunction among women is 34.3%, and it was related to age, educational level, age at menopause, and frequency of sex (9).

There is also evidence indicating that sexual function may be negatively impacted by anxiety (10). Fatigue, lack of sex drive, and poor lubrication are important factors commonly experienced by breastfeeding women which may cause sexual dysfunction and anxiety (11). In addition, evidence has shown that anxiety affects the breastfeeding initiation and duration, and exclusive breastfeeding (12).

Interventions such as cognitive behavioral therapy, yoga, music therapy, massage, relaxation, and counseling are methods to reduce anxiety during pregnancy and postpartum (13). Rose damascene is a species of Rosaceae family flowers widely planted in different provinces of Iran (14). Farnia et al. found that Rose damascene could significantly increase sexual desire, orgasm, and sexual satisfaction in women who suffered from depression and took antidepressant medication (15).

A systematic review including 32 articles showed that Rose damascene can significantly reduce state anxiety, depression, and stress, but does not affect the trait anxiety (16).

Despite the few studies conducted on the effect of Rose damascene on sexual function and anxiety, but there is still scarce data regarding breastfeeding women. Therefore, this study was conducted to evaluate the effect of Rose damascene on anxiety and sexual function of breastfeeding of women.

## 2 Methods

This was a randomized controlled trial involving 94 breastfeeding women. The protocol of the study was approved by the Ethics Committee of Ahvaz Jundishapur University of Medical Sciences (ref no: IR.AJUMS.REC.1400.614). The protocol of the study was also registered in the Iranian Registry of Randomized Controlled Trial (ref. no: IRCT20211015052775N1, Registration date: 14/02/2022).

### 2.1 Inclusion/exclusion criteria

Women with following characteristics were recruited: breastfeeding, having passed 6 weeks to 18 months from delivery, having a husband, being primiparous with singleton baby, having basic literacy, and obtaining a score < 26 form Female Sexual Function Index (FSFI questionnaire) and score 20–53 from Spielberger questionnaire.

Women with following criteria were excluded from the study: allergy to rose flower and its derivations, using medications (e.g., anti-depressants) affecting sexual function, feeding baby with baby formula, smoking and alcohol drinking, pregnancy, history of any mental illness or severe psychological trauma in the last 6 months.

### 2.2 Sample size

According to a previous study (17), the following formula was used to calculate the sample size:


$$\begin{array}{l} n=\frac{{\left(Z_{1-\frac{\alpha }{2}}+Z_{1-\beta }\right)}^{2}\left(s_{1}^{2}+s_{2}^{2}\right)}{{\left(d\right)}^{2}}\\ \;=\frac{{\left(1.96+0.84\right)}^{2}\left(3.03^{2}+3.47^{2}\right)}{{\left(1.95\right)}^{2}}\approx 42 \end{array}$$

In this formula, the power of study was 80%,

(*α*) = 0.05, *β* = 0.2, S1 = 3.03, S2 = 3.47, d = 2

We added 10% for attrition and the total number of samples rose to 94 and 47 in each group.

### 2.3 Randomization and allocation concealment

Block randomization technique with a block size of 4 and an allocation ratio of 1:1 was used for randomization. Randomization plan was prepared by a statistician (EM).

The Rose damascene extract and placebo capsules were placed in similar packages and distributed among participants. Two labels (A and B) were chosen for Rose damascene extract and placebo, and neither the researcher nor the participants were aware group allocation.

### 2.4 Drug preparation

At first, the flowers were harvested from gardens. Then they were dried and ground under standard conditions. Afterwards, the extract was prepared using ethanol 70% after 48 h maceration method. The extract was then passed from a filter paper, and after the hydroalcoholic solution was separated from the plant and using the rotary machine, the remaining ethanol was collected. The extract was dried and stored at 37°C. The dried extract was later stored in −20°C to make the capsules. The thick and very viscous extract of the rose was mixed with starch by the granulation method and sieved with a mesh size of 1700 μm to form granules. Then the granules were dried for one and a half hours at 65°C and passed through another sieve with a mesh size of 710 μm to make them suitable for encapsulation by homogenizing the granules. The produced capsules contained 400 mg Rose damascene.

The placebo was made using starch and placed in 400 mg capsules that were identified to Rose damascene capsules. Rose damascene and placebo were prepared in the Pharmacology School of Yasuj University of Medical Sciences, Iran.

### 2.5 Setting

A public health center in Yasuj, Iran was considered for data collection. The data collection started in May 2023 and ended in September 2023.

### 2.6 Intervention

After written consent was obtained from eligible participants, they were randomly assigned into two groups of Rose damascene and placebo. The women received 56 capsules of Rose damascene or placebo to take for four weeks (two capsules per day). After four weeks, they received the second round of intervention including 56 capsules to be taken for four weeks. The women were requested to complete the FSFI and Spielberger questionnaire prior to the intervention, and eight weeks after it. The reminder was sent by phone every other day to all participants by one of the researchers (GA). The participants were requested to record any side effect of the drugs or placebo. They could also call one of the researchers (GA) in case of any problem. Although, we could not find any study on the safety of Rose damascene among breastfeeding women, other studies reported no side effects of this extract among pregnant women during labor and delivery (18, 19).

### 2.7 Instruments

A demographic questionnaire, an obstetric questionnaire, the Female Sexual Function Scale (FSFI), and the Spielberger questionnaire were used to collect the data.

The demographic questionnaire consisted of questions about age, husband’s age, education, husband’s education, weight, height, occupation, marriage duration, and support from family and husband. The content validity of this questionnaire was assessed and confirmed. The obstetric questionnaire included data about mode of delivery, type of feeding, and the onset of sexual relationship after delivery.

The FSFI includes 19 questions in six dimensions: sexual desire (two questions), sexual arousal (four questions), lubrication (four questions), orgasm (three questions), sexual satisfaction (three questions), and pain (three questions). The scores of each domain were multiplied in certain factor (0.6 for sexual desire, 0.3 for arousal and lubrication, 0.4 for orgasm, sexual satisfaction and pain). The questions of each domain were scored from almost never (1) to almost always (5). The minimum and maximum scores of this questionnaire were 2 and 36, respectively. Scores more than 26 represent good sexual function (20). The psychometric evaluation of the Persian version of FSFI questionnaire was done in Iran by Fakhri et al. (21).

The Spielberger State–Trait Anxiety questionnaire includes 40 questions developed by Spielberger et al. in 1970. This questionnaire measures both state anxiety (20 questions) and trait anxiety (20 questions). Each question is scored from almost never (1) to almost always (4). The minimum and maximum scores of this questionnaire ranged from 20 to 80 (22). The psychometric evaluation of this questionnaire in Iran has been done by Abdoli et al. (23).

The participants were weighed using a digital scale (Seca, Germany) while wearing light cloths and standing bare-footed. The participants’ height was measured using a stadiometer (Seca, Germany) while standing bare-footed. The body mass index (BMI) was calculated by dividing weight (kg) to height (m2).

### 2.8 Outcomes

Sexual function and anxiety.

### 2.9 Follow-up

Participants were evaluated regarding anxiety and sexual function before the intervention and eight weeks after that.

### 2.10 Statistical analyses

All data were entered into SPSS version 22. Quantitative variables were reported as mean, standard deviation, and minimum and maximum, while qualitative variables were reported as number and percentages. Chi-square test or Fisher’s exact test was used to investigate the relationship between qualitative variables, whereas independent t-test or its non-parametric equivalent (Mann–Whitney test) was used to compare the two groups in terms of quantitative variables. To determine the effectiveness of the intervention during the study period, the analysis of variance (ANCOVA) with adjustment for confounding factors and before intervention was used. *p* < 0.05 was considered statistically significant.

## 3 Results

**Figure 1:**
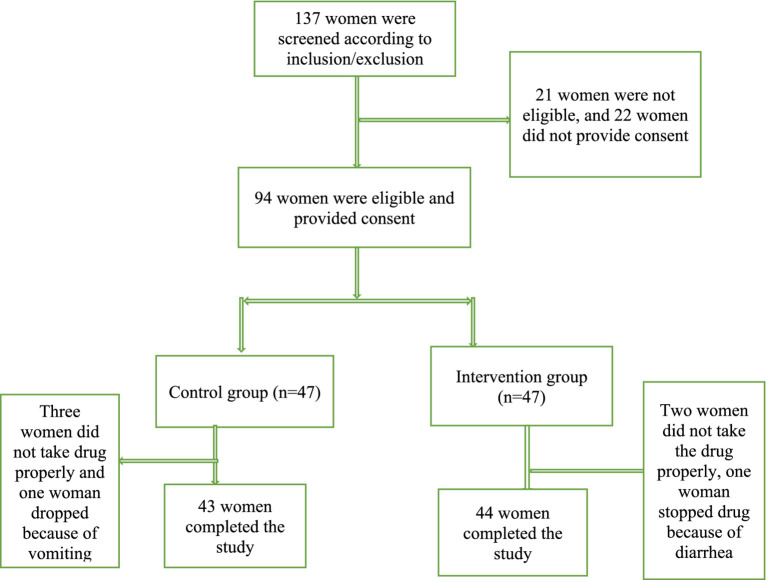
Recruitment and retention of participants in the study.

In this study, 94 women were recruited, of whom, 87 completed the study (44 in the intervention group and 43 in the control groups, Figure 1). Table 1 shows the demographic and obstetric characteristics of the participants in the two groups. The mean ± SD of the participants’ age was 28.05 ± 7.39 and 28.58 ± 5.82 years in the intervention and control groups, respectively. Most of the women in the intervention group (*n* = 20; 45.5%) had a high school diploma, while in the control group, the majority (*n* = 23; 53.5%) had university education (*p* = 0.039). Most women in the two groups had a moderate economic status and were housewives. The two groups did not significantly differ regarding demographic variables except for the level of education. With regard to obstetrics information, most of the participants had undergone cesarean section (50 and 58.1% in the intervention and control groups, respectively). Most women resumed their sexual activity <2 months after childbirth. Women in the control group received significantly more emotional support from their husband compared to the control group (93% vs. 75%, *p* = 0.022). The women in the two groups were not statistically different in terms of other obstetrics variables.

**Table 1:** Demographic and obstetric characteristics of the participants in the intervention and control groups.

Variables	Intervention *n* = 44	Control *n* = 43	*p* value
	Mean ± SD		
Age (y)	28.05 ± 7.39	28.58 ± 5.82	9.462
Age of husband (y)	32.55 ± 6.25	33.12 ± 5.47	0.943
Body mass index (kg/m2)	25.50 ± 4.78	27.25 ± 3.74	0.835
	*N* (%)		
Education			
Primary	11 (25)	4 (9.3)	0.039
High school	20 (45.5)	16 (37.2)	
University education	13 (29.5)	23 (53.5)	
Education of husband			
Primary	3 (6.8)	6(14)	0.53
High school and diploma	19 (43.2)	16(37.2)	
University education	22(50)	21(48.8)	
Economic status			
Weak	19 (43.2)	10(23.3)	0.105
Moderate	22(50)	31(72.1)	
Good	3 (6.8)	2 (4.7)	
Occupation			
Housewife	39 (88.6)	37 (86)	0.716
Employed	5 (11.4)	6(14)	
Time passed after delivery			
6 weeks to 6 months	7 (15.9)	13 (30.2)	0.248
6 months to 12 months	28(63.6)	21 (48.8)	
12–18 months	9 (20.5)	9 (20.9)	
Mode of delivery			
Vaginal + episiotomy	18(40.9)	16(37.2)	
Vaginal without episiotomy	4 (9.1)	2 (4.7)	
Cesarean section	22 (50)	25 (58.1)	
Onset of sexual relationship after delivery			
<2 months	29 (65.9)	33 (76.7)	0.377
2–6 months	10 (22.7)	9 (20.9)	
≥6 months	4 (9.1)	1 (2.3)	
Psychological support of husband			
Yes	33 (75)	40 (93)	0.02
No	11 (25)	3 (7)	
Psychological support of family			
Yes	38 (86.4)	41 (95.3)	0.147
No	6 (13.6)	2 (4.7)	

Table 2 shows the components of sexual function in intervention and control groups before and after the intervention. Before intervention, the mean score of sexual desire was 3.04 ± 0.86 and 3.37 ± 0.79 in the intervention and control groups, respectively, which improved significantly in the intervention group after intervention (4.26 ± 0.63 vs. 3.39 ± 0.0.81, respectively, *p* < 0.001).

**Table 2:** The sexual function components in the intervention and control group before and after intervention.

Variables	Intervention *n* = 44	Control *n* = 43	Test value	*P* value	Effect size
	Mean ± SD				
Sexual desire					
Before intervention	3.04 ± 0.86	3.37 ± 0.79	46.995	<0.001	0.367
After intervention	4.26 ± 0.63	3.39 ± 0.0.81			
Sexual arousal					
Before intervention	2.98 ± 1.11	3.36 ± 0.97	37.14	<0.001	0.314
After intervention	4.27 ± 0.49	3.48 ± 0.94			
Lubrication					
Before intervention	2.97 ± 0.97	3.53 ± 0.76	19.97	<0.001	0.198
After intervention	4.19 ± 0.64	3.68 ± 0.79			
Orgasm					
Before intervention	3.10 ± 1.14	3.41 ± 0.89	28.34	<0.001	0.259
After intervention	4.27 ± 0.52	3.63 ± 0.93			
Sexual satisfaction					
Before intervention	4 ± 1.19	4.03 ± 1.08	51.72	<0.001	0.39
After intervention	5.11 ± 0.61	4.02 ± 0.96			
Pain					
Before intervention	3.50 ± 0.96	3.73 ± 0.59	17.23	0.0004	0.175
After intervention	3.09 ± 0.41	3.60 ± 0.63			
Total score of sexual function					
Before intervention	19.61 ± 5.02	21.46 ± 3.08	57.13	<0.001	0.41
After intervention	25.21 ± 1.62	21.82 ± 3			

For all analyses, ANCOVA was used after adjusting for education, psychological support of husband, and the score of all dimensions of sexual function before the intervention.

The mean score of sexual arousal was 2.98 ± 1.11 and 3.36 ± 0.97 in the intervention and control groups, respectively, which improved significantly in the intervention group (*p* < 0.001). The mean score of lubrication was 2.97 ± 0.97 and 3.53 ± 0.76 (*p* < 0.05) in the intervention and control groups, respectively, which improved significantly in the intervention group (*p* < 0.001).

As far as orgasm was concerned, the mean score was 3.10 ± 1.14 and 3.41 ± 0.89 in the intervention and control groups, respectively before the intervention. This score rose significantly in the intervention group compared to the control group (4.27 ± 0.52 vs. 3.63 ± 0.93, *p* < 0.001). The two groups were significantly different in terms of their sexual satisfaction score before intervention (4 ± 1.19 vs. 4.03 ± 1.08), but there was a significant improvement significantly in the intervention group compared to the control group after intervention (5.11 ± 0.61 vs. 4.02 ± 0.96, *p* < 0.001). Regarding the mean score of pain, there was no significant difference between the two groups before intervention (3.50 ± 0.96 vs. 3.73 ± 0.59), but after the intervention, this score reduced significantly in the intervention compared to the control group (3.09 ± 0.41 vs. 3.60 ± 0.63, *p* = 0.0004). Finally, the total score of sexual function was 19.61 ± 5.02 and 21.46 ± 3.08 in the intervention and control groups, respectively, which improved significantly in the intervention group as opposed to the control group (25.21 ± 1.62 vs. 21.82 ± 3, *p* < 0.001).

Table 3 shows the scores of trait and state anxieties in the two groups before and after the intervention. As evident from this table, the score of trait anxiety was 47.97 ± 4.68 and 48.44 ± 5.89 in the intervention and control groups, respectively before intervention. After the intervention, however, this score improved significantly in the intervention group compared to the control group (51.63 ± 3.53 vs. 48.13 ± 5.57, *p* < 0.001).

**Table 3:** Trait and state anxiety in the intervention and control groups before and after intervention.

Variables	Intervention *n* = 44	Control *N* = 43	Test value	*P* value	Effect size
	Mean ± SD				
Trait anxiety					
Before intervention	47.97 ± 4.68	48.44 ± 5.89	17.15	<0.001	0.175
After intervention	51.63 ± 3.53	48.13 ± 5.57			
State anxiety					
Before intervention	45.75 ± 8.98	45.72 ± 8.51	0.725	0.397	0.0009
After intervention	43.31 ± 6.41	44.30 ± 8.87			

For all analyses ANCOVA was used with adjusting for education, psychological support of husband, and the score of state and trait anxiety before the intervention.

The two groups did not have any significant difference regarding state anxiety (45.75 ± 8.98 vs. 45.72 ± 8.51) before intervention, and although this score improved in the intervention group compared to the control group after the intervention, the difference was not significant (43.31 ± 6.41 vs. 44.30 ± 8.87, *p* = 0.397).

One woman in the intervention group dropped-out because of diarrhea and one woman in the control group reported vomiting.

## 4 Discussion

This study was designed to evaluate the effect of Rose damascene on anxiety and sexual function in breastfeeding women. Our results showed that the mean scores of sexual dimensions including sexual desire, arousal, lubrication, orgasm, satisfaction, and pain as well as total score of sexual function improved in the group receiving Rose damascene for 8 weeks compared to the control group. One of the mechanisms of the effect of Rose damascene on sexual function may be attributed to the activation of post-synaptic 5-hydroxytryptamin (5-HT), which is receptor of raphe nuclei in midbrain (24). Also, the effect of Rose damascene on sexual function may be explained by its flavonoids, and anthocyanins components (25). An animal study showed that damask rose extract can significantly increase the levels of FSH, LH, and testosterone compared to the control group, which means that this extract can stimulate the hypothalamic–pituitary axis (26).

In a study on 80 women in reproductive age, Motaharinezhad et al. found that eight weeks of intervention including rose oil could significantly improve the sexual function of the studied women (27).

In another study conducted on 60 patients who had selective serotonin reuptake inhibitors-induced sexual dysfunction, Farina et al. found that rose oil could significantly reduce depression and improve sexual function of male participants (28). The results of the present study are in line with those of Motaharinezhad et al. and Farina et al. In the present study, although the total scores of FSFI increased in the intervention group, it remained under the cut-off point for normal sexual function. This could be explained by the fact that the sexual function score of Iranian women is usually lower than that in other countries (29).

Our results also showed that Rose damascene could significantly reduce the trait anxiety in the intervention group, while the differences between the two groups regarding state anxiety were not statistically significant. The mechanism of how rose oil reduces anxiety and stress may be contributed to the effect of this oil on reduction of cortisol, as attested by a study by Fukui et al. who found that rose oil could significantly reduce the level of cortisol in healthy individuals (30). Also, Hogratanaworakit found that rose oil can decrease the number of breaths, and systolic blood pressure, and result in relaxation in participants who received this oil (31). Furthermore, Fukada et al. found that inhalation of rose extract by rats that were under the stress elevated the level of corticosterone, and in turn, reduced the level of stress (32).

We could not find any study on the effect of rose extract on anxiety, but Emadikhalaf et al. found that four weeks of aromatherapy using rose scent resulted in reduced job stress among nurses (33).

### 4.1 Strengths and limitations of the study

This is the first study to evaluate the effect of Rose damascene on anxiety and sexual function of breastfeeding women in Iran. Despite its merits, this study has some limitations. First, talking about sexual issues in Iranian culture is a taboo, and participants may not express their real feelings about sexuality. Second, FSFI questionnaire measures sexual function of participants in the past month, and thus results may be affected by recall bias. Finally, the education and psychological support of husband was significantly lower in the intervention group compared to the control group, however, the ANCOVA test was used for adjusting these two variables, and the score of all dimensions of sexual function before the intervention.

## 5 Conclusion

The results of present study showed that Rose damascene could significantly improve the sexual functions of breastfeeding women. It also decreased the trait anxiety but failed to improve the state anxiety. Using this herb is recommended for increasing the sexual function of breastfeeding women.

## Data Availability

The raw data supporting the conclusions of this article will be made available by the authors, without undue reservation.
